# Diagnosis of Perihilar Osteoclast Giant Cell Carcinoma in an Elderly Patient: A Report of Diagnostic Challenges Over Several Attempts

**DOI:** 10.14309/crj.0000000000001829

**Published:** 2025-09-15

**Authors:** Sharifeh Almasaid, Fathima Keshia Suhail, Nesrine Bensaadi, Kelita Singh, Bishnu Sapkota

**Affiliations:** 1 Department of Internal Medicine, State University of New York Upstate Medical University Hospital, Syracuse, NY; 2 Department of Internal Medicine, Division of Gastroenterology, State University of New York Upstate Medical University Hospital, Syracuse, NY; 3 Department of Pathology, State University of New York Upstate Medical University Hospital, Syracuse, NY

**Keywords:** Cholangiocarcinoma, undifferentiated carcinoma, osteoclast-like giant cells, perihilar region, cholangioscopy

## Abstract

Cholangiocarcinoma is a rare gastrointestinal malignancy with an annual incidence of 0.3 to 6 cases per 100,000, and, usually, it is reported as adenocarcinoma in about 95% of the cases. It can develop as a result of combination of genetic predispositions and various risk factors like but not limited to choledochal cysts, cholelithiasis/choledocholithiasis, yet its diagnosis is challenging due to difficulties in obtaining tissue for diagnosis. In this study, we present a challenging case in diagnosis that reveals undifferentiated carcinoma with osteoclast-like giant cells in the perihilar region of the bile duct.

## INTRODUCTION

Cholangiocarcinoma (CCA) is a rare and aggressive gastrointestinal (GI) malignancy originating from the biliary epithelium, accounting for approximately 3% of GI cancers.^1,2^ It is classified into intrahepatic and extrahepatic forms, with the latter further divided into perihilar, hilar, and distal cholangiocarcinoma, depending on the tumor's location relative to the cystic duct insertion.^3^

The global incidence of CCA has been rising, reported at 0.3 to 6 cases per 100,000 individuals annually, with higher prevalence in Asian countries.^4,5^ This increase is attributed to a combination of genetic predispositions and various risk factors, including a choledochal cysts, cholelithiasis/choledocholithiasis, chronic pancreatitis, inflammatory bowel diseases, and primary sclerosing cholangitis.^6,7^ Its diagnosis is challenging, largely due to difficulties in obtaining tissue for histological confirmation. Histopathology typically shows that adenocarcinoma comprises about 95% of CCA cases.^1^

This raises critical questions: Howstrongly should hilar biliary lesions be suspected as cholangiocarcinoma? What is the optimal diagnostic modality, given that tissue acquisition can be challenging and may require multiple procedures. In this study, we present an 80-year-old man with obstructive jaundice, who underwent multiple endoscopic evaluations and biopsies to diagnose his perihilar osteoclast giant cell carcinoma.

## CASE REPORT

**Figure 1.**
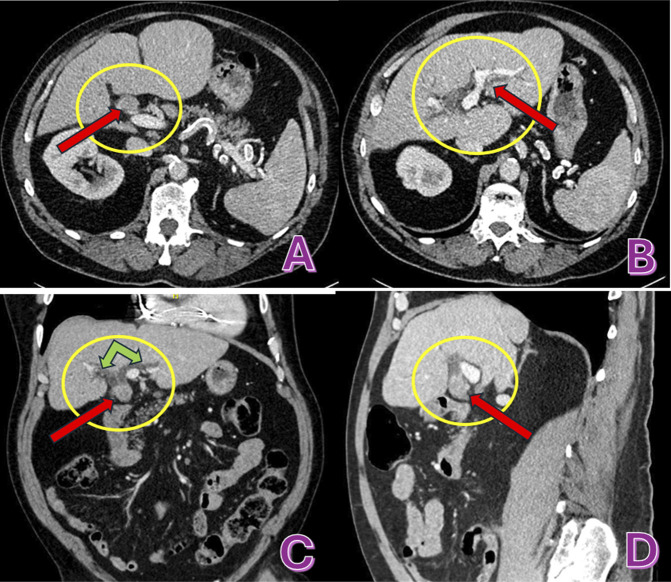
(A) Axial view portal venous phase shows the homogenously enhancing mass, immediately anterior to the portal vein without evidence of portal vein occlusion. (B) Axial view portal venous phase, showing central intrahepatic ductal dilation due to the mass effect. (C) Coronal view, Portal venous phase images, shows a homogenously enhancing, soft tissue attenuating nodular opacity or mass at the proximal common hepatic duct (red arrow), with upstream dilation of the right and left main hepatic ducts (green arrows). (D) Sagittal view, portal venous phase, shows the homogenously enhancing mass with the same findings as above.

**Figure 2.**
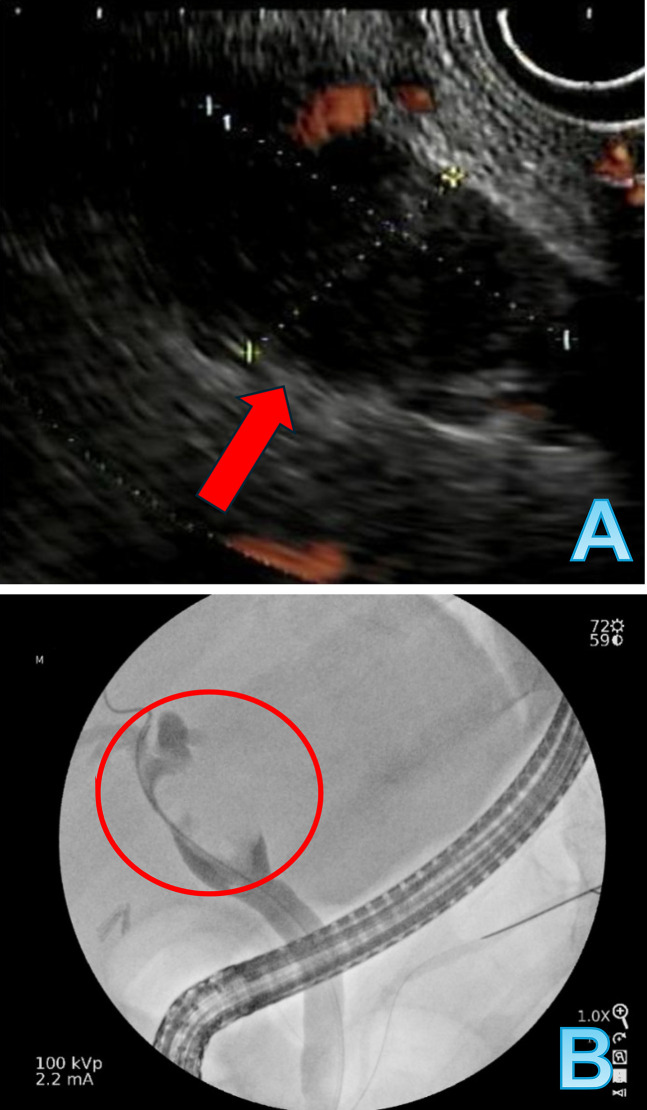
(A) EUS showing hypoechoic mass in the CHD. (B) ERCP showing a filling defect in the common hepatic duct, proximal end of the mass was just below or at the level of bifurcation.

An 80-year-old man with ileocolonic Crohn's disease, compensated metabolic dysfunction-associated steatohepatitis cirrhosis, and cholelithiasis s/p cholecystectomy presented with dark-colored urine for 4 weeks followed by pale-colored stool for 1 week. Laboratory results demonstrated aspartate aminotransferase 38 IU/L, alanine transaminase 57 IU/L, alkaline phosphatase 245 IU/L, and total bilirubin of 1.6 mg/dL that reflects cholestatic pattern with concern for bile duct obstruction. Computed tomography with contrast of the abdomen and pelvis revealed a 1.7 × 2.1 × 1.9 cm rounded mass at common hepatic duct (CHD) just below the bifurcation with moderate intrahepatic biliary ductal dilatation (Figure 1). Of note, magnetic resonance imaging performed a year ago for abnormal liver enzymes, did not reveal biliary masses or ductal dilation. Given the evidence of a perihilar mass on computed tomography, the decision was made to proceed with endoscopic ultrasound (EUS)/endoscopic retrograde cholangiopancreatography (ERCP) evaluation as the patient had a pacemaker that was not compatible with the magnetic resonance imaging machine in the facility. EUS showed a hypoechoic mass with subtle vascularity in the CHD. The proximal end of the mass was just below or at the level of bifurcation measuring 25 mm by 15 mm in greatest diameter. The CHD in this area was dilated to 16-17 mm in diameter (Figure 2). ERCP revealed a large, irregular filling defect at CHD extending to just below the bifurcation consistent with EUS findings (Figure 2). Biliary cannulation was achieved, bile was aspirated, brushings were sent for cytology, and a plastic biliary stent was placed. The cytology was negative for malignancy. Tumor markers were sent after for workup completion; he was found to have elevated CA19-9 at 81 and normal carcinoembryonic antigen level of 0.6.

**Figure 3.**
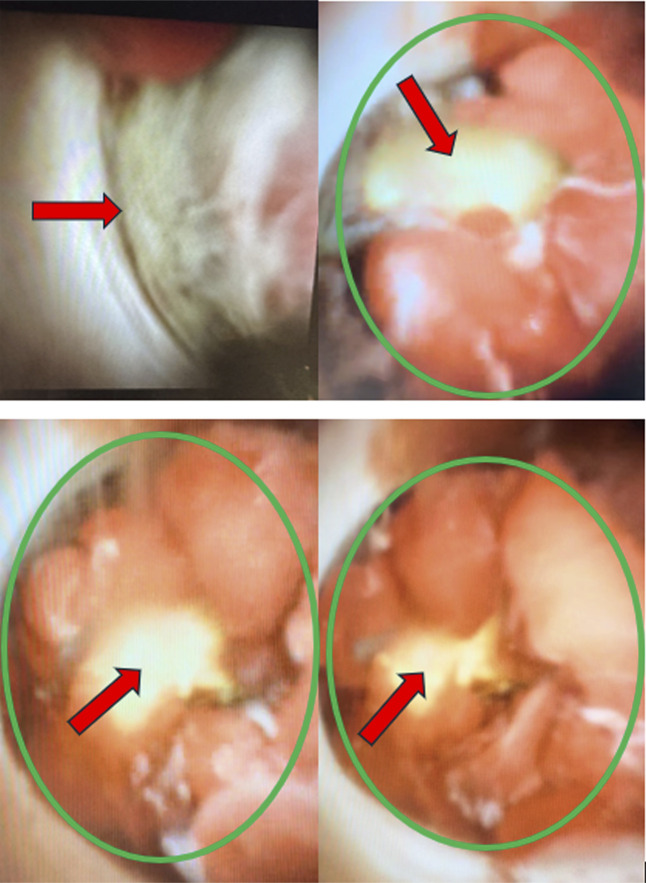
Cholangioscopy showing a mass (green circle) noted upon advancement to perihilar area with cholesterol deposit (red arrow) and tissue component.

Before his next scheduled ERCP for stent exchange, the patient presented with a concern for stent occlusion with elevated LFTS, aspartate aminotransferase 49, alanine transaminase 57, total bilirubin 5, direct bilirubin 3.8, and ALK 330. Repeat ERCP with cholangioscopy was performed revealing an occluding mass at the CHD just below the bifurcation (Figure 3). Neoplastic suspicion remained high as the mass was easily bleeding, and blood clots were seen in the bile duct. Biopsies were obtained using SpyBite forceps along with bile aspirate, and then a plastic stent was placed into the bile duct across the stricture. Pathology showed acute and chronic inflammation, with no evidence of malignancy.

Given high suspicion for cancer, the patient was brought back for repeat EUS, ERCP, and cholangioscopy. The hilar mass was sampled using SpyBite, brushing, and bile was aspirated and sent for evaluation. EUS fine-needle aspiration (FNA) was not performed due to concern for bile leak and seeding if it is malignant. EUS evaluation also revealed peripancreatic nodes near the pancreatic head that were sampled with FNA for cytology and biopsy. The cytology and pathology from the mass was reported as atypical cytology, unable to rule out malignancy. The lymph nodal FNA was benign.

**Figure 4.**
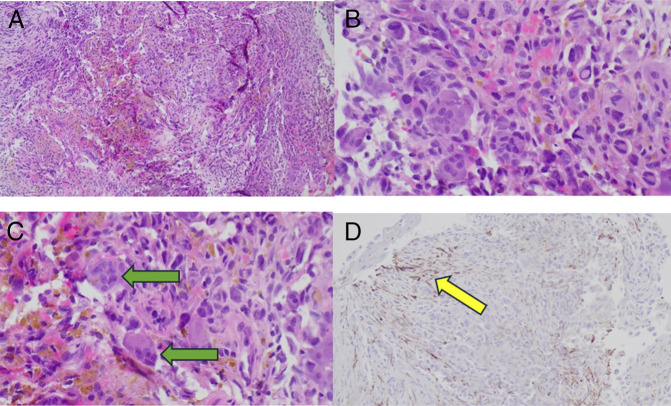
Highly pleomorphic tumor cells (A), with increased mitotic activity (B) and scattered osteoclast-like giant cells (green arrows) (C). (D) Shows immunohistochemical staining with AE1/AE3 positivity in the spindle cells (yellow arrow).

The patient presented within a week after his procedure with obstructive jaundice and a dislodged stent. ERCP and cholangioscopy were performed; mass was identified again from which biopsies and brushing were obtained. These samples were positive for malignancy, evident by sheets of single and multinucleated highly pleomorphic tumor cells with increased mitotic activity, histiocytic infiltrate with hemosiderin deposition, and scattered osteoclast-like giant cells. Immunohistochemical staining showed AE1/AE3 positivity in the spindle cells, but the sample lacked biliary differentiation given negative CK7 testing. Also, metastatic colorectal origin was ruled out as the sample was negative for CK20 and CDX2. The final diagnosis was undifferentiated carcinoma with osteoclast-like giant cells (UCOGC) (Figure 4).

The patient was referred to a transplant center for possible surgical resection; staging scans were performed and indicated a potentially respectable tumor. He decided against surgical resection and wanted to follow with hematology/oncology. Treatment was delayed 3 months after diagnosis due to small bowel obstruction and recurrent obstructive jaundice. He started induction chemotherapy with a combination of gemcitabine and cisplatin; immunotherapy was not used initially due to the history of Crohn's. He completed 6 cycles of combination chemotherapy, and in the sixth cycle, pembrolizumab was added to the regimen and, currently, he is receiving it with 200 mg every 3 weeks.

## DISCUSSION

Undifferentiated carcinoma is a malignant epithelial tumor that mimics sarcoma and has different compositions of spindle, polygonal, and giant cells.^8^ It was proposed that bone marrow-derived mononuclear histiocytes/macrophages drawn to the tumor by chemotactic signals generated by the neoplastic cells, fuses to form osteoclast-like giant cells.^9^ Juan Rosai was the first to describe 2 cases of pancreatic tumor that were nearly identical to giant cell tumor of the bone in 1968, after which the concept of extra skeletal osteoclast-like giant cell tumors in the alimentary tract was established.^10^

Though still rare, multiple examples of undifferentiated carcinoma with osteoclast-like giant cells (UCOGC) in the GI tract have been documented. The pancreas is the most frequently reported site, but it has also been seen in the liver, small intestine, ampulla of Vater, and common bile duct.^11–18^

A total of 6 cases have described UCOGC involvement in CBD, 4 were in the distal portion, 1 case in middle, and 1 was involving the entire CBD and CHD. The age range was 56-81 years, male-to-female ratio was 2:1, and the clinical presentation varied from asymptomatic to symptomatic with jaundice and abdominal pain. Surgery was the mainstay of treatment depending on the location, where these cases were managed with Whipple procedures, pylorus-preserving pancreatic head resection and pancreaticogastrostomy, excision of the gallbladder and extrahepatic bile duct with a Roux-en-Y hepaticojejunostomy, or by cholangioscopic tissue removal. Four of 6 cases were free of disease after surgical intervention; however, the other 2 cases were lost to follow-up.^19^

Our patient had an obvious mass in the CHD just below the bifurcation, and direct cholangioscopy-guided biopsy would yield a definite result. Since brush cytology alone has a low diagnostic yield (40%) if malignancy is suspected, we chose multimodality sampling to boost sensitivity in this instance. Adding fluoroscopic-guided biopsy samples can raise the yield to 60%.^20,21^ EUS was used to assess perihilar mass and regional lymph nodes. FNA of mass was avoided as it was near the hilum, and we wanted to avoid needle seeding. SpyBite biopsy was used because it is superior to traditional endoscopic diagnostic techniques.^20,21^

Cholangioscopy has several drawbacks despite all these advantages. There are challenges obtaining a good tissue sample given the small size of SpyBite biopsies that may fail to capture malignant cells and the requirement for several sampling attempts, as what happened in our case and let to delay in diagnosis. Furthermore, there is a chance of complications from the procedure, including cholangitis, pancreatitis, and perforation. Its usefulness is further restricted financially by the expenses of the apparatus, such as the processor and disposable scopes, as well as the need for general anesthesia.^22,23^

Surgery has been regarded as the first line of treatment with the best possibility of curing the disease, although there is currently no definite radiological description, prognosis, or standardized treatment due to its rarity. Although neoadjuvant chemotherapy has been proposed as a potentially helpful strategy in patients with borderline-resectable tumors, its significance in the management of UCOGC is yet unknown.^24^ Regarding adjuvant therapy, treatment regimens are similar to those used for pancreatic adenocarcinoma are typically applied. These include FOLFIRINOX regimens for patients who are fit and gemcitabine-based regimens for those who are frailer. In addition, the use of anti-PD-1 antibody therapy has shown efficacy in treating lung metastases, highlighting the potential benefits of immunotherapy for these tumors.^25^ Instead of having surgery, our patient chose to get chemotherapy with cisplatin and gemcitabine, followed by pembrolizumab.

To the best of our knowledge, our case presents the first case of CHD bifurcation involvement that was challenging and required multiple endoscopic evaluations and biopsies for final diagnosis of UCOGC. The high initial suspicion of malignancy meant the patient was followed very closely and had a low threshold for repeat endoscopic evaluation. In conclusion, not all biliary masses are cholangiocarcinomas; therefore, broader differentials in addition to multimodal biopsy techniques with short interval follow-up should be considered for such lesions.

## DISCLOSURES

Author contributions: S. Almasaid: Collecting information, drafting the manuscript. FK Suhail: Drafting the manuscript and revising. N. Bensaadi: Obtaining Pathology slides and description. K. Singh and B. Sapkota: Revising the manuscript. S. Almasaid is the article guarantor.

Financial disclosure: None to report.

Informed consent was obtained for this case report.

## ABBREVIATIONS

CCA: cholangiocarcinoma
CHD: common hepatic duct
ERCP: endoscopic retrograde cholangiopancreatography
EUS: endoscopic ultrasound
FNA: fine-needle aspiration
GI: gastrointestinal
UCOGC: undifferentiated carcinoma with osteoclast-like giant cells
